# Resting-State Network Topology Differentiates Task Signals across the Adult Life Span

**DOI:** 10.1523/JNEUROSCI.2406-16.2017

**Published:** 2017-03-08

**Authors:** Micaela Y. Chan, Fahd H. Alhazmi, Denise C. Park, Neil K. Savalia, Gagan S. Wig

**Affiliations:** ^1^Center for Vital Longevity, School of Behavioral and Brain Sciences, University of Texas at Dallas, Dallas, Texas, 75235 and; ^2^Department of Psychiatry, University of Texas Southwestern Medical Center, Dallas, Texas, 75390

**Keywords:** aging, connectivity, dedifferentiation, networks, resting-state, task activity

## Abstract

Brain network connectivity differs across individuals. For example, older adults exhibit less segregated resting-state subnetworks relative to younger adults (Chan et al., 2014). It has been hypothesized that individual differences in network connectivity impact the recruitment of brain areas during task execution. While recent studies have described the spatial overlap between resting-state functional correlation (RSFC) subnetworks and task-evoked activity, it is unclear whether individual variations in the connectivity pattern of a brain area (topology) relates to its activity during task execution. We report data from 238 cognitively normal participants (humans), sampled across the adult life span (20–89 years), to reveal that RSFC-based network organization systematically relates to the recruitment of brain areas across two functionally distinct tasks (visual and semantic). The functional activity of brain areas (network nodes) were characterized according to their patterns of RSFC: nodes with relatively greater connections to nodes in their own functional system (“non-connector” nodes) exhibited greater activity than nodes with relatively greater connections to nodes in other systems (“connector” nodes). This “activation selectivity” was specific to those brain systems that were central to each of the tasks. Increasing age was accompanied by less differentiated network topology and a corresponding reduction in activation selectivity (or differentiation) across relevant network nodes. The results provide evidence that connectional topology of brain areas quantified at rest relates to the functional activity of those areas during task. Based on these findings, we propose a novel network-based theory for previous reports of the “dedifferentiation” in brain activity observed in aging.

**SIGNIFICANCE STATEMENT** Similar to other real-world networks, the organization of brain networks impacts their function. As brain network connectivity patterns differ across individuals, we hypothesized that individual differences in network connectivity would relate to differences in brain activity. Using functional MRI in a group of individuals sampled across the adult life span (20–89 years), we measured correlations at rest and related the functional connectivity patterns to measurements of functional activity during two independent tasks. Brain activity varied in relation to connectivity patterns revealed by large-scale network analysis. This relationship tracked the differences in connectivity patterns accompanied by older age, providing important evidence for a link between the topology of areal connectivity measured at rest and the functional recruitment of these areas during task performance.

## Introduction

Functionally distinct brain areas exhibit distinct patterns of connections (Felleman and Van Essen, 1991). The activity and connectivity of a brain area are not mutually exclusive, as they often constrain one another. For example, neural recording in both nonhuman primates and felines has demonstrated how the specific inputs and outputs associated with a given brain area define the range of its possible functions (Arieli et al., 1995; Tsodyks et al., 1999). Research on connectivity has largely focused on mapping distinctions in connectivity across brain areas to differences in their function (Ungerleider and Desimone, 1986). However, the connectivity patterns of brain areas (topology) exhibit between-subject variability as well (Markov et al., 2011). It is largely uncertain whether and how such intersubject differences in connectivity affect brain function; the present report examines this important question.

Noninvasive imaging has enabled researchers to examine the link between areal connectivity and function in the human brain. Clustered patterns of connectivity measured at rest [resting-state functional correlations (RSFCs); Biswal et al., 1995] have been shown to overlap with task-evoked activation maps (Power et al., 2011; Yeo et al., 2011; Tavor et al., 2016). Whereas the correspondence between RSFC patterns and task-evoked activation maps helps to elucidate the processing roles of particular brain areas, graph theory has provided a formal framework to model the brain as a large-scale network by simultaneously evaluating the connections between multiple distributed brain areas (Bullmore and Sporns, 2009; Wig et al., 2011). Network models of RSFC patterns have revealed the presence of functionally distinct communities (modules/subnetworks), some of which align with known brain systems (e.g., default system, visual system; Petersen and Sporns, 2015). Variations in the RSFC patterns of areas and communities have been linked to differences in task-evoked brain activity (Cole et al., 2013; Bertolero et al., 2015), aligning with earlier animal work that demonstrated how distinctions in connectivity relate to distinctions in processing demands.

The evidence thus far has established a link between RSFC connectivity and task-related activity. It is uncertain whether and how differences in large-scale brain network organization across individuals moderates functional activity. One prominent and common source of variation in both brain network organization and functional activity has been observed across the healthy adult life span (Grady et al., 1994; Park et al., 2004; Betzel et al., 2014; Chan et al., 2014; Geerligs et al., 2015). Here, we leverage age-related variability in brain network organization and task activity to (1) understand how variation in the connectional topology of brain areas may relate to variation in task-related activity across multiple task- and stimulus-processing demands and (2) determine whether this topology–function relationship is stable across individuals of different ages with documented differences in brain network organization.

We focus on an important distinction in nodes that is imposed by community organization, which is thought to reflect functional differences. The presence of communities results in two types of nodes with distinct connectional properties: “connector” nodes serve as “bridges” between communities, whereas “non-connector” nodes predominantly exhibit connections to nodes within their own community. Non-connector nodes are often viewed as being specialized for performing particular processes—a community's functional focus can be defined by the specialization of its non-connector nodes. Connector nodes exhibit diverse interactions with various communities, resulting in broader roles involving information integration. Consistent with these ideas, observations across various complex systems have demonstrated important differences in how connector and non-connector nodes process and disseminate information (Guimerà et al., 2005; Deng et al., 2012; Harriger et al., 2012). Does information processing revealed by task activity respect a node's distinct role in brain networks? How does this relationship alter across the adult life span, which is accompanied by changes in brain network organization? Functional activity may be agnostic to individual differences in connectional topology. If patterns of connectivity are related to function however, then topological distinctions of brain areas may be a guiding framework for understanding variation in functional activity across subjects, or vice versa.

## Materials and Methods

### Participants

Participants were recruited as part of the Dallas Lifespan Brain Study (DLBS) from the Dallas–Fort Worth community and provided written consent before participating. All study procedures were reviewed and approved by the institutional review boards at the University of Texas at Dallas and the University of Texas Southwestern Medical Center. A total of 266 participants completed a resting-state fMRI scan. Following a rigorous data screening and cleaning procedure (see below for details of preprocessing and quality control), data from 238 participants were available for analysis in the final sample (see Table 1).

**Table 1.** Demographic information of final sample

Age groups (years)	*N*	% female	Mean education years (SD)
YA (20–34)	64	59%	16.44 (2.37)
ME (35–49)	51	65%	16.15 (2.17)
ML (50–65)	46	67%	16.98 (2.05)
OA (65–89)	77	60%	16.18 (2.38)

### Imaging data acquisition

The DLBS collects a range of imaging modalities including a T1-weighted structural MRI scan and seven functional MRI (fMRI) blood oxygenation level–dependent (BOLD) scans. The fMRI scans a resting-state scan with one run, and two experimental tasks, each with a different number of runs. All brain scans were acquired with a Philips Achieva 3T whole-body scanner and a Philips 8-channel head coil at the University of Texas Southwestern Medical Center using the Philips SENSE parallel acquisition technique.

#### Structural MRI (MPRAGE)

A T1-weighted sagittal magnetization-prepared rapid acquisition gradient echo structural image was obtained (TR, 8.1 ms; TE, 3.7 ms; flip angle, 12°; FOV, 204 × 256 mm; 160 slices with 1 × 1 × 1 mm voxels). The scan duration was 3 min and 57 s.

#### Functional MRI

All BOLD scans were collected with the following parameters: TR, 2000 ms; TE, 25 ms; flip angle, 80°; FOV, 220 mm; 43 interleaved axial slices per volume; 3.5/0 mm (slice thickness/gap); in-plane resolution, 3.4 × 3.4 mm. For each functional run, five additional volumes were collected at the beginning of each scan to allow for T1 stabilization. These five volumes were discarded during preprocessing.

##### Word judgment scan (semantic task).

Participants completed one block-design run (231 volumes) of the semantic task. Participants viewed 128 words and judged whether each word referred to a living or nonliving object. Participant's responses were entered using a button box in their right hand. Half of the stimuli were unambiguously living or nonliving (e.g., horse, truck), whereas the other half of the stimuli were ambiguous and harder to classify as living or nonliving (e.g., virus, sponge). Words were organized into blocks such that there were eight unambiguous blocks and eight ambiguous blocks, with each block containing eight words. Within a block, each word was displayed for 2.5 s, followed by a fixation cross for 0.5 s. All stimuli were presented in lowercase, white font at the center of a black screen. A fixation cross was displayed for 6 s between each block. In the present study, ambiguous and unambiguous word conditions were modeled together as a “words” condition.

##### Scene classification scans (visual task).

Participants completed three event-related design runs (171 volumes each) of the visual task. Participants viewed colored images of outdoor landscapes and had to determine whether there was water present in the scene (e.g., lake, river, ocean). Participants' responses were entered using a button box in their right hand. Within each run, 32 images were presented for 3 s each (jittered with an interstimulus interval ranging from 4 to 14 s, during which time a white fixation cross was centrally presented on a black background). All picture stimuli were obtained from a previously published study (Gutchess et al., 2005) and presented at the center of a black screen. Half of the stimuli (48 total across three runs) were scenes containing water in the images. Although participants completed a surprise recognition test outside of the scanner on these scene stimuli, the results of this memory test are not incorporated into the present analysis. In the present study, all pictures were modeled together to form a “visual” condition. A mechanical error occurred during the collection of the visual task for one participant, resulting in one less data point for analyses involving the visual task.

##### Resting-state scan.

Participants completed one run (154 volumes) of an eyes-open fixation resting-state scan. Participants were instructed to stay still and fixate on a white crosshair centrally presented on a black screen for the duration of the scan. The experimenter verified that participants complied with the instructions and did not fall asleep during the functional scan via verbal confirmation.

### Preprocessing

#### Structural MRI preprocessing

FreeSurfer 5.3 was used to process volumetric images into cortical surface images. This process includes brain extraction, segmentation, generation of white matter and pial surfaces, inflation of the surfaces to a sphere, and surface shape-based spherical registration of the participant's “native” surface to the fsaverage surface (Dale et al., 1999; Fischl et al., 1999a; Ségonne et al., 2005).

Automated FreeSurfer outputs were first visually inspected for poor skull stripping, inclusions of vessels or other tissue that neighbor the cortex, and insufficient intensity normalization obscuring the gray and white matter boundary. Manual editing and rechecking were completed as needed to account for the inaccuracies in the automated outputs (Savalia et al., 2017).

A single deformation map for each individual was generated by combining the (1) deformation map created when registering an individual's native surface to FreeSurfer's fsaverage atlas and (2) the deformation map for registering fsaverage-aligned data to a hybrid left–right fsaverage surface (fs_LR; Van Essen et al., 2012). Each individual's native FreeSurfer-generated output was then registered to fs_LR using the single deformation map in a one-step resampling procedure.

#### Standard fMRI preprocessing

All BOLD images (task and resting-state) were processed to reduce artifacts. Images were corrected for (1) odd versus even slice intensity differences attributable to interleaved acquisition without gaps and (2) head movement within and across runs. Realignment was completed within each task (across multiple runs) by estimating the transformation matrix for each functional frame relative to the first frame (of the first run if there are multiple runs).

#### RSFC preprocessing

Additional preprocessing steps were used to reduce spurious variance unlikely to reflect neuronal activity in RSFC data: (1) Multiple regression of the BOLD data was performed to remove variance related to the whole brain (global) signal, ventricular signal, white matter signal, and their derivatives (six signal regressors derived from eroded FreeSurfer masks), the six detrended head realignment parameters obtained by rigid body head motion correction of the current frame (*t*) and the previous frame (*t* − 1), and the squared estimates of *t* and *t* − 1 motion parameters (the “Friston24” motion regressors; Friston et al., 1996). (2) Bandpass filtering (0.009–0.08 Hz) was performed. (3) Temporal masks were created to flag motion-contaminated resting-state frames based on frame-by-frame displacement (Power et al., 2014), calculated as the sum of absolute values of the differentials of the three translational motion parameters (*d_ix_*, *d_iy_*, *d_iz_*) and three rotational motion parameters (α*_i_*, β*_i_*, γ*_i_*). Resting-state frames with frame displacement (FD) >0.3 mm were removed. (4) The removed frames were replaced by interpolated data to insure that artifacts from discarded frames did not blur into the remaining data (Carp, 2013). (5) Steps 1 and 2 were repeated on the resting-state time series with interpolated data. (6) The interpolated frames were removed in the final resting-state time series, which was used for correlation calculation. If <75 frames of data were available after frame removal (scrubbing), the participant was discarded from further analyses (see Table 1 for the number of participants in each age group after scrubbing). Across the entire sample, the mean number of frames kept after scrubbing was 139 (min, 75; max, 154). While the number of frames kept differed across cohorts (*F*_(3,234)_ = 4.789, *p* < 0.001), the results presented in the present manuscript are qualitatively similar when frames analyzed were equated across participants or when FD was included as a covariate in the statistical models where age is a predictor.

Including global signal regression as a part of the processing stream has been an issue of considerable debate given the possibility of removing genuine neural signals that are embedded in the global signal (Schölvinck et al., 2010). However, considerable evidence has now demonstrated that a major component of the global signal includes spatially nonspecific signal artifacts, in which head motion can play a significant role (Satterthwaite et al., 2013; Power et al., 2014, 2017). As documented by both others and us, older adults exhibit greater amounts of head movement (Mowinckel et al., 2012; Van Dijk et al., 2012; Savalia et al., 2017), which has been shown to systematically alter the correlation structure of resting-state signals (Power et al., 2012; Satterthwaite et al., 2013; Yan et al., 2013; Zeng et al., 2014). The minimization of this source of bias was thus viewed as a priority to prohibit erroneous interpretation in any cross-subject or cross-cohort comparison, especially when the variable of interest (in our case, age) was systematically related to head motion (*r*_age×mean FD_ = 0.422, *p* < 0.001). It has been demonstrated that at present, this is best achieved via regression of the global signal (Power et al., 2014).

### Mapping functional data to surfaces

Preprocessed resting-state data and fMRI task β volumes were registered to the fs_LR (32 k) left and right hemisphere surfaces because of evidence of better alignment of cortical anatomy when compared to linear or nonlinear volume-based registration (Fischl et al., 1999b). The previously constructed single deformation map for each individual was used for one-step resampling of the functional data to the fs_LR surfaces. For each participant, functional data from the volumetric gray matter ribbon were mapped to the individual's native surface mesh and then deformed to the fs_LR surfaces using predefined single deformation map. Data were smoothed across the surface using a Gaussian smoothing kernel (σ = 2.55). Critically, transforming participants' functional data into fs_LR surface space enables time course and β weight extraction from nodes that were defined and generated on the same surface (Wig et al., 2014).

### fMRI task analysis

fMRI task data were analyzed using the general linear model (GLM) in SPM 8 (Wellcome Department of Cognitive Neurology; http://www.fil.ion.ucl.ac.uk/spm/). For each task, the durations of each block/event and runs were modeled, along with nuisance regressors (linear trends to model signal drift, constants to model mean signal intensity in a run, and the six head realignment parameters to model head movement). The β maps of each condition of interest were then surface mapped (for details, see above, Mapping functional data to surfaces) to allow direct comparison with surface-mapped RSFC data. Region-of-interest statistical analyses of task data were performed on the mean fMRI β values extracted from each of the putative area centers (349 disks; see below, Functional area node identification) from the surface-mapped β maps. Nodes within the default system were excluded from analyses related to task activation to avoid conflation of patterns of task-evoked activation and deactivation (Shulman et al., 1997). Results in the present manuscript remain qualitatively similar when default system nodes were included in all analyses.

### Brain graph construction

Surface-mapped resting-state fMRI data were analyzed using a modification of a previously published node set (Chan et al., 2014), whereby a node-by-node correlation matrix (i.e., brain graph) was constructed for each participant.

#### Functional area node identification

Brain graphs were constructed using a modified set of previously published nodes (Chan et al., 2014). To construct these nodes, the locations of putative area centers were first identified in a published surface-based RSFC boundary map generated using data from younger adults (Wig et al., 2014). To minimize locations of parcellation uncertainty (at the parcellation map boundaries or borders), we focused on the local minima of the boundary maps (i.e., centers of putative areas). A total of 441 area centers were identified across the two hemispheres, with a criterion of being at least 8 mm apart. Disks of 3 mm radius were created around each of these surface locations to create nodes representing areal locations.

It has been pointed out that signal intensity may be poorer in certain locations of the brain (e.g., anterior and inferior portions of the temporal and orbital frontal cortex), potentially leading to lower quality of parcellation in these areas (Wig et al., 2014). As nodes derived from these locations may be inaccurately defined and/or exhibit unreliable signals, nodes in locations of lower BOLD signal were identified and discarded using the mean BOLD signal intensity map used to create the original parcellation (Wig et al., 2014, their Fig. 8). After normalizing the whole brain signal with a mode value of 1000 (Ojemann et al., 1997), nodes in locations with mean BOLD signal intensity below 800 were discarded; this resulted in a final node count of 349 across the two hemispheres.

It is worth noting that the node set was created from an independent data set of young adults (Wig et al., 2014). While it is possible that area parcellation may differ across the healthy adult life span (Wig et al., 2012; Han et al., 2016; with preliminary work showing little significant differences), considerable emphasis was placed on ensuring cross-participant alignment of anatomical features using surface-based registration. This process followed an iterative procedure of extensive manual quality checks and editing in combination with automated brain segmentation using FreeSurfer (Savalia et al., 2017). As an additional step, areas were modeled using nodes representing small disks localized to the putative centers of brain areas as opposed to entire patches, given the greater likelihood for an area's border to exhibit across-participant variation relative to its more central locations (Wig et al., 2011).

#### Edge definition: preparing RSFC data for connectivity analysis

For each participant, the resting-state fMRI time series of vertices within each of the predefined nodes was extracted and the vertex-mean time course was computed for each node. The cross-correlation of each node's time course with every other node's time course was incorporated into a node-to-node correlation matrix. Last, correlation coefficients were converted into *z*-values using Fisher's formula (Zar, 1996), resulting in the final Fisher's *z*-transformed *r* matrix (*z*-matrix) for each participant. The *z*-matrix is a fully connected, weighted relatedness graph. Although negative edges have been shown to be useful in characterizing certain network properties (Rubinov and Sporns, 2011), due to ambiguity in interpreting negative correlations that followed necessary regression of global signals as per the arguments above (Murphy et al., 2009; Gotts et al., 2013; e.g., possible introduction of negative correlation), negative edges were excluded from the *z*-matrices before all analyses, in accordance with our previous study (Chan et al., 2014).

### Identifying reliable age-group-specific functional systems

#### Infomap community detection

Community detection is a class of algorithms that specialize in finding clusters/groups of similar objects in a graph. Age-group-specific community assignments for brain nodes were determined by applying the Infomap community detection algorithm on participant-averaged *z*-matrices of each age group (Rosvall and Bergstrom, 2008; Fortunato, 2010).

#### Obtaining reliable age-group-specific community assignments

The present study focused on capturing regular patterns across both graph edge densities (2–10%) and also participants within an age group. Quantifying reliable assignments in this way minimizes the likelihood of representing the potentially atypical graph properties driven by a single individual or only present at a particular edge density. A bootstrap approach was used to generate reliable community assignments for each age group across 2–10% edge densities with the following steps: (1) resampling with replacement was used to create 1000 bootstrapped age-group mean *z*-matrices for each age group. (2) These bootstrapped mean *z*-matrices were thresholded across 2–10% edge densities, creating 1000 matrices at each edge density, for each age group. (3) Community detection was performed on each thresholded bootstrapped mean matrix, resulting in 1000 community assignments for each age group, at each edge density. The community assignments were labeled based on their overlap with a set of published RSFC functional systems (Power et al., 2011). Last, (4) at each edge density, the most common assignment across the 1000 community assignments was selected as the node's reliable assignment.

Several systems were not included in the association system categorization due to too few node counts across multiple edge densities (e.g., salience system; superior temporal gyrus) or being near areas of poor signal (e.g., inferior temporal pole, ventral frontal pole).

### Defining node-level connectivity and identifying system-specific connector and non-connector nodes

As used in previous studies of brain area connectivity (Power et al., 2013; Cole et al., 2013; Bertolero et al., 2015), the participation coefficient (PC) was used to quantify the extent to which a node is connected to other systems proportional to its overall connection through the following formula (Guimerà and Nunes Amaral, 2005b; Rubinov and Sporns, 2010): PC*_i_* = 1 − Σ_*m*∈*M*_[*k_i_^w^*(*m*)/*k_i_^w^*]^2^, where *k_i_^w^*(*m*) is the weighted connections of node *i* with nodes in system *m* (a system that node *i* does not belong to), and *k_i_^w^* is the total weighted connections node *i* exhibits. A higher PC for a given node indicates proportionally greater connectivity with nodes in other systems relative to that node's total connections. The PC of each node was calculated based on age-group-specific community assignments in each of the 2–10% edge density matrices (see above, Obtaining reliable age-group-specific community assignments).

Due to higher PCs in association systems than sensory–motor systems, a PC threshold across the entire network would categorize the majority of the nodes in associations systems as connector nodes instead of distinguishing the connector and non-connector nodes within each system. Therefore, nodes were classified as connectors or non-connectors based on the median PC of nodes within their system for each age group. A node was labeled as a connector if its PC was greater than the median PC of nodes in their own system, and as a non-connector if its PC was less than or equal to the median PC of nodes in their own system. While we focus here on the superordinate categorization of connector versus non-connector node types in the present study, we also note that a finer distinction can be made among non-connector nodes that discriminate provincial hubs (i.e., non-connector nodes with many connections within their own system) from peripheral nodes (Guimerà et al., 2005).

Where appropriate, PC was also treated as a continuous variable (i.e., without categorizing nodes into connector and non-connectors). In analyses involving PCs (i.e., both those treating PC as a continuous variable and those using PC to define connector status), a node's PC was first calculated at each edge density (2–10%) and then these were summed together to account for variations of graph configuration across edge densities.

### Computing activation selectivity

For each participant, a selectivity score was calculated based on the PC and β activation across the participant's nodes. The selectivity score was quantified to determine how a node's topology was associated with its task activity using the following formula: selectivity = −1 × *r*(PC, β), where *r*(PC, β) is the correlation coefficient across all nodes of PC and β estimates during a task. The coefficient was multiplied by negative one to generate a score where a positive score reflected greater activation among nodes with lower PCs (non-connector nodes), and a negative score reflected greater activation among nodes with higher PCs (connector nodes). A score close to zero represents the absence of a relationship between the nodes' RSFC-defined topology and BOLD activity during task performance.

## Results

### The connectional topology of areal nodes at rest differentiates task-evoked activation

**Figure 1.**
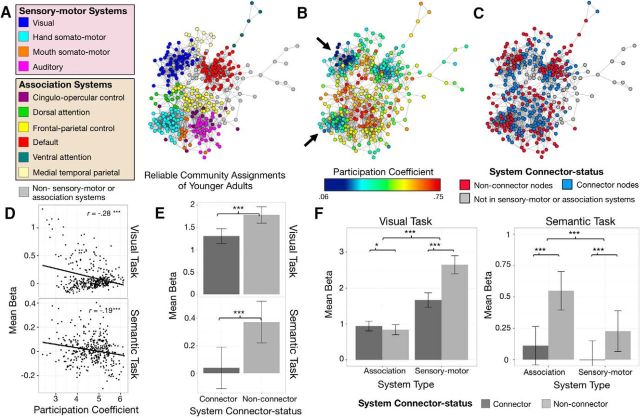
Node topology as defined by brain connectivity at rest relates to activation selectivity as a function of task demands. ***A***, Reliable community assignments (corresponding to distinct functional brain systems) of younger adults at 4% edge density. ***B***, PC of each node showing sensory–motor systems (e.g., visual and hand somatosensory system; black arrows) generally exhibited lower PCs than association systems (e.g., frontal–parietal control system at the center of the spring-embedded graph). ***C***, RSFC-defined connector status of nodes at 4% edge density, defined within each system. The majority of the nodes with fewer connections to other systems (non-connector nodes in red) are situated along the periphery of the network. Nodes that are not part of our categorization of sensory–motor and association systems are colored gray in spring-embedded graphs depicting community assignments and connector status. ***D***, Nodewise correlation between each node's overall connectedness to other systems (defined by PC, calculated across 2–10% edge densities) and its task-evoked functional activation (defined by mean β values) in both tasks revealed that nodes with lower connectedness generally exhibited greater activation. ***E***, RSFC-defined non-connector nodes exhibited greater task activation than RSFC-defined connector nodes in both visual and semantic tasks. ***F***, During the visual task, activation in non-connector nodes was greater than activation in connector nodes of sensory–motor systems but not association systems. Conversely, during the semantic task, greater activation of non-connector versus connector nodes was observed both types of systems, but the effect was greater in the association systems. All error bars represent SEs. *p < 0.05; *** *p* < 0.001.

Young adult participants' (*n* = 64, age = 20–34y) brain networks were constructed using a node set consisting of 349 disks (3 mm radius) built around putative area centers across the cortical surfaces of the two hemispheres (see Materials and Methods for details on node definition and brain graph construction). Edges between all node pairs were derived by Fisher *z*-transformed Pearson correlations that were calculated from their resting-state time series. Reliable community assignments were first generated with Infomap community detection using a bootstrap approach that labeled the node set with community assignments across 2–10% edge densities. Community detection identifies highly interconnected groups of nodes that are segregated from other groups of nodes; in the brain network, a number of these communities correspond to distinct brain systems that are hypothesized to subserve distinct domains of information processing (e.g., visual processing, task-level control). Accordingly, for the remainder of this report, the term “community” will be used when describing a general network property, whereas the term “system” will be used when describing a functionally labeled community in the brain. At a broader level of description, we examined distinctions in activity and connectivity between two types of systems: sensory–motor and association systems (Fig. 1*A*). Sensory–motor systems can be broadly categorized as those systems that are engaged in neural coding and transformation of incoming sensory and outgoing motor information, whereas association systems are those systems that typically direct and integrate information in a wide range of tasks and across multiple modalities (Mesulam, 1990; Posner and Petersen, 1990).

The connector status of a node was operationalized by the node's participation coefficient (Guimerà and Nunes Amaral, 2005a; Rubinov and Sporns, 2010), which represents a node's proportion of connections to nodes in other communities relative to its total number of connections. Nodes identified with higher PCs typically serve as connectors between communities. The functional activity of each node was measured by calculating mean β estimates from GLMs applied to two independent fMRI tasks. These tasks activated distinct sets of brain areas and largely reflected different domains of cognitive processing: (1) a scene classification task (visual task), during which participants judged whether pictures of outdoor scenes contained water, and (2) a word judgment task (semantic task), which required participants to determine whether a presented word referred to a living or nonliving entity.

In general, nodes with lower PCs exhibited greater activity, during both tasks (*r*_visual_ = −0.277, *p* < 0.001; *r*_semantic_ = −0.193, *p* < 0.001; Fig. 1*D*). While a node's PC is a continuous measure, it can be used to create a categorical distinction between connector nodes and non-connector nodes within each of the functional systems (Guimerà and Nunes Amaral, 2005a; Rubinov and Sporns, 2010). The median PC of each system was used to categorize nodes as either connector nodes (node PC > median system PC) or non-connector nodes (node PC ≤ median system PC; Fig. 1*B*,*C*). In contrast to previous studies (Power et al., 2013; Yeo et al., 2015), this categorization was implemented within each system as opposed to across the entire brain network, thus allowing us to distinguish and examine the topological and functional differences of nodes relative to other nodes in their own independent systems. RSFC-defined non-connector nodes exhibited greater task-related activity across both the visual and semantic tasks (*F*_(1,62)_ = 113.78, *p* <. 001; *post hoc t* tests of each task, visual task, *t*_(62)_ = −7.65, *p* < 0.001; semantic task, *t*_(63)_ = −10.53, *p* < 0.001; Fig. 1*E*). Critically, however, this effect exhibited specificity with respect to the type of processing linked to each of the brain systems and task demands. An ANOVA model examining task-evoked activity as predicted by connector status (connector vs non-connector node), type of fMRI task (visual vs semantic), and system type (sensory–motor vs association systems) revealed that the magnitude of task activity (BOLD signal β estimate) was significantly predicted by all three main effects (*F*_(1,62)_ > 33.06, *p* values ≤0.001; greater activity in non-connector nodes, the visual task, and sensory–motor systems). Importantly, the main effects were qualified by a significant three-way interaction (*F*_(1,62)_ = 64.00, *p* < 0.001), which demonstrated that the greater activation of non-connector nodes (i.e., activation selectivity) was specific to the systems that were most relevant to each of the tasks (Fig. 1*F*). To highlight the preferential task activation of nodes based on their connectional topology, for the remainder of this report we refer to the differential activation of non-connector relative to connector nodes as activation “selectivity.”

During the visual task, sensory–motor systems exhibited greater activation selectivity (non-connector vs connector, *M*_β_ = 2.65 vs 1.67; *t*_(62)_ = −7.79; *p* < 0.001) than the association systems (non-connector vs connector, *M*_β_ = 0.84 vs 0.94; *t*_(62)_ = 2.61; *p* = 0.011). Follow-up analyses revealed that this effect was prominent in the visual system (non-connector vs connector; *M*_β_ = 6.02 vs 3.71) but not in other sensory–motor systems (i.e., hand somatosensory, mouth somatosensory, auditory; non-connector vs connector, *M*_β_ = 0.15 vs 0.13). Conversely, for the semantic task, association systems exhibited greater activation selectivity (non-connector vs connector, *M*_β_ = 0.55 vs 0.11; *t*_(63)_ = −10.34; *p* < 0.001) than the sensory–motor systems (non-connector vs connector, *M*_β_ = 0.23 vs −0.01; *t*_(63)_ = −5.19; *p* < 0.001). Unlike the visual task, the semantic task's effect of activation selectivity was not confined to a specific association system. *Post hoc* analyses of individual systems revealed that multiple association systems exhibited greater activation in non-connector nodes than connector nodes (e.g., frontal parietal system, ventral attention system, cingulo-opercular system, dorsal attention system, and medial temporal parietal system; *t*_(63)_ < −3.33; *p* ≤ 0.001).

### Topological distinctions between connector and non-connector nodes decrease with increasing age

The preceding observations suggest that both a node's system membership (i.e., whether it is in one of the sensory–motor systems or in one of the association systems) and topological position within its system (e.g., connector vs non-connector) are directly related to the nodes' activation profile during performance of goal-directed tasks. If the connector status of a node is an important feature of network topology, differences in network organization that have a bearing on dissociating connector and non-connector nodes should impact the differentiation of task signals across individuals.

To directly test this prediction, we examined the relationship between network organization and task activity in a sample of participants that have been shown to exhibit robust variations in the organization of their brain networks (Chan et al., 2014). Older age is accompanied by both decreases in connectivity within brain systems and increases in connectivity between systems. These patterns manifested as decreased system segregation in older age. We incorporated the RSFC data from adult participants across the life span to examine age-related differences in the distribution of connector and non-connector nodes and determine whether these differences may relate to variations in task-evoked activity. The full sample included 238 health adults ranging from 20 to 89 years old: younger adults (YAs), *n* = 64; age, 20–34 years; middle early adults (MEs), *n* = 51; age, 35–49 years; middle late adults (MLs), *n* = 46; age, 50–64 years; older adults (OAs), *n* = 77; age, 65–89 years.

**Figure 2.**
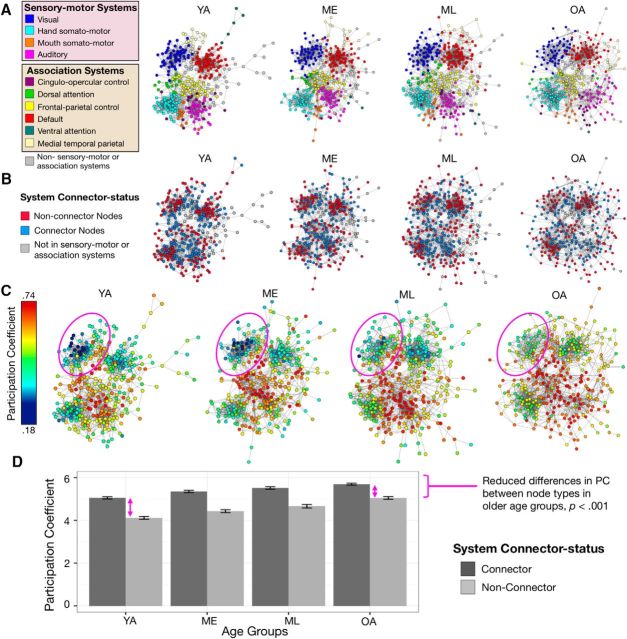
Increasing age is associated with reduced distinctions between RSFC-defined connector and non-connector nodes. ***A***, Spring-embedded graphs depicting the community assignments of YA, ME, ML and OA at 4% edge density demonstrate that a collection of prominent functional systems is detectable in each age group. ***B***, RSFC-defined connector status of nodes was classified within each age group relative to their affiliated system's median PC. ***C***, Within systems, connector and non-connector nodes exhibit reduced differences in their PCs with increasing age (circles). ***D***, Direct comparison of a node's PC (calculated across 2–10% edge densities) as a function of node type highlights significantly decreasing differences in connector versus non-connector nodes with increasing age. Error bars represent SEs.

The presence of reliable community assignments was largely similar across age, with the same prominent functional systems consistently identified within each of the age groups (Fig. 2*A*). Despite the broad similarities in system organization across the four age groups, distinctions between connector and non-connector nodes decreased with increasing age (Fig. 2*B*,*C*). An ANOVA model examining PC (mean across 2–10% network edge densities) in relation to age group (YA, ME, ML, OA) and connector status (connector vs non-connector) revealed that while older adults generally exhibited greater mean node PCs than younger adults, non-connector nodes showed greater age-related increases in their PCs compared to connector nodes, yielding an interaction of age group by connector status: *F*_(3,234)_ = 24.89, *p* < 0.001 (Fig. 2*D*). The interaction occurred as a result of diminished distinctions in the connectional topology between connector and non-connector nodes among older adults compared to younger adults (as displayed in Fig. 2*C*).

### The selective activation of non-connector nodes decreases with increasing age

**Figure 3.**
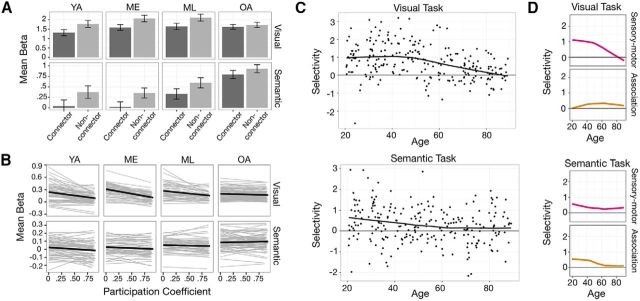
Age-related differences in activation selectivity in relation to task demands and system type. ***A***, Across the four age groups, non-connector nodes exhibited greater activity than connector nodes in both tasks, with diminished differences observed in OAs. Error bars represent SEs. ***B***, Nodewise correlation between PC and β (calculated at 2–10% edge densities) within each individual revealed generally negative relations, with older adults exhibiting a weaker PC–β relation. Data at different densities are presented as separate regression lines. ***C***, Younger adults exhibited positive selectivity score [−1 × *r*(PC, β)], reflecting greater BOLD activation among nodes with lower PCs, where this effect approaches zero (no preferential activation of either type of node) in older adults. ***D***, The age-related differences in selective activation were observed more prominently in the sensory–motor systems during the visual task (top), and in the association systems during the semantic task (bottom). In ***A*** and ***B***, *y*-axes of the two tasks are scaled differently to enable better display of the differences between connector and non-connector nodes during the semantic task.

How do the differences in connectional topology of brain areas observed across healthy aging impact their recruitment during goal-directed tasks? Based on the preceding observations, one possibility is that the decreasing connectional distinctiveness between connector and non-connector nodes in older ages results in decreasing activation selectivity during task performance. Alternatively, the differences in connectional topology at rest may have no relevance to task activation in older age. An ANOVA model predicting task-evoked activity (mean β estimates) by node type (connector and non-connector nodes), task (visual vs semantic), and age group revealed a significant three-way interaction. Consistent with the hypothesis that connectional topology is related to function, greater activation selectivity as a function of connectional topology was found for younger compared to older age groups, across both tasks (age group × task × connector status, *F*_(1,233)_ = 3.36, *p* = 0.020; Fig. 3*A*)

This form of activation selectivity as a function of node topology can be further visualized and quantified on a participant-by-participant basis. In addition, to further highlight the specificity of the observed relationship and differences across age, we correlated each individual's nodes' PCs with their corresponding BOLD activity. As expected, non-connector nodes exhibited greater task-evoked activity, and this “selectivity” diminished in older participants (Fig. 3*B*). To quantify activation selectivity in relation to connectional topology, a selectivity measure was computed for each participant by correlating the RSFC-defined PCs and BOLD activity of nodes; greater selectivity scores reflect greater activation in nodes with low PCs (see Materials and Methods for a description of the calculation of selectivity score). As expected from the significant main effect of connector status in the previous ANOVA, the mean selectivity score for both tasks was positive on average [*M*_visual_ = 0.73 (SD, 0.93) and *M*_semantic_ = 0.30 (SD, 0.88)], and increasing age was negatively correlated with selectivity (*r*_visual_ = −0.36, *p* < 0.001; *r*_semantic_ = −0.19, *p* = 0.003; for trends fitted with locally weighted scatterplot smoothing (LOESS) curve, see Fig. 3*C*).

**Figure 4.**
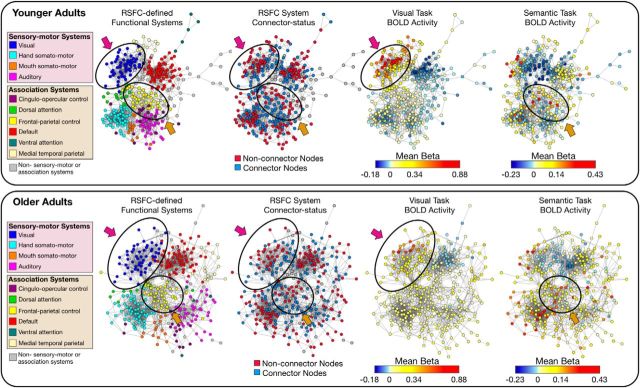
Older adults exhibit reduced activation selectivity in relation to differences in node connectivity patterns. Spring-embedded graphs (4% edge density) depict the (1) RSFC-defined community assignments, (2) RSFC-defined node connector status (system specific), (3) mean BOLD activity during the visual task, and (4) mean BOLD activity during the semantic task in both younger (top) and older (bottom) age groups. Two distinct functional brain systems [the visual system (blue in leftmost panels) and the frontal–parietal control system (yellow in leftmost panels)] are circled to highlight the differences in activation selectivity as a function of task demands, system type, and age. Younger adults exhibit greater activity in non-connector compared to connector nodes in the visual system (magenta arrows) and frontal–parietal control system (orange arrows) during performance of the visual task and semantic task, respectively. This activation selectivity is reduced in older adults. Interestingly, the differences in activity between connector and non-connector nodes are not limited to activations, but are apparent in differences in task-evoked deactivations as well (task-induced deactivation is greater in younger adults).

We established earlier how the activation selectivity of specific tasks is constrained to the functional systems that mediate the processing demands of the task (Fig. 1*F*). There is evidence that age-related topological differences vary as a function of system type (i.e., sensory–motor vs association systems; Chan et al., 2014). If the functional recruitment of areas during a given task is indeed related to the connectional topology of those areas that are important for the task's processing requirements, there should be a direct relationship between the variations in a node's connectional topology and its task-evoked activity across participants. To address this, selectivity scores for each participant's sensory–motor and association systems were calculated separately for each task. A GLM predicting selectivity scores by age (continuous), system type (sensory–motor vs association), and task (visual vs semantic) yielded a significant three-way interaction (age × task × system type, *F*_(1,235)_ = 26.35, *p* < 0.001). Age-related differences in selectivity of connectional topology were primarily related to the systems relevant to the task demands. *Post hoc* analyses revealed that age-related differences in selectivity during the visual task were observed in the sensory–motor systems but not association systems (*r*_sensory–motor_ = −0.385, *p* < 0.001 vs *r*_association_ = 0.013, *p* = 0.846; Fig. 3*D*). Conversely, age-related differences in selectivity during the semantic task were significant in the association systems but not sensory–motor systems (*r*_sensory–motor_ = −0.105, *p* = 0.106 vs *r*_association_ = −0.256, *p* < 0.001). The results highlight the specificity of the relationship between age-related differences in RSFC-defined node topology as a function of system type and selectivity of task activation, and can be further appreciated in the visualization of brain network connectivity and activation patterns presented in Figure 4.

## Discussion

Investigations of brain organization and information processing typically focus on structure and function at specific spatial scales (e.g., areas, systems) or individual features (e.g., task-evoked activity, connectivity). The present findings offer evidence for a relationship between a brain area's topological organization at rest and its functional recruitment during task performance. In a sample of younger adults, we first demonstrated that the topology of a given node as defined by RSFC network patterns predicted the node's magnitude of task-evoked activity. RSFC-defined non-connector nodes, identified by relatively greater predominance of connections to nodes within their own functional system relative to other nodes of the same system, exhibited greater brain activity than connector nodes defined within each system. This selective activation was specific to task-relevant systems. To test whether this relationship persists across networks with different topological organization and areal recruitment, we extended our analyses to include participants representing the adult life span from ages 20–89 years; these participants had previously been shown to exhibit differences in their RSFC network topology (Chan et al., 2014). We found reduced topological distinctions between system-specific connector and non-connector nodes in adults with increased age. The reduced topological distinctions observed in older adults correspond to reduced differentiation of connector versus non-connector nodes during task performance. The results provide evidence that network topology observed at rest may play a role in constraining functional activity of brain areas. Furthermore, the findings provide a novel network-based hypothesis to explain numerous observations that have highlighted a “dedifferentiation” of brain activity prominently observed in aging cohorts of individuals (Grady et al., 1994; Cabeza, 2002; Park et al., 2004).

### The connectional topology of nodes relate to their function

In many real-world networks, a node's function is largely determined by its patterns of connections (Guimerà et al., 2005; Harriger et al., 2012). In brain networks, there is evidence that nodes that serve as bridges between communities or systems may be functionally different from nodes with a preponderance of connections to nodes within their own system. For example, across the entire brain network, brain areas (nodes) that exhibited many connections to areas in systems other than their own were shown to be functionally more diverse, exhibiting greater probability to activate during a large variety of tasks (Bertolero et al., 2015). Consistent with this, brain systems exhibiting greater connectivity across the entire brain (e.g., associative and control systems; Cole et al., 2013) are more likely to be engaged during cognitive tasks that place different processing demands on distinct functional systems, suggesting greater functional flexibility for areas in these association systems (Yeo et al., 2015). In these prior reports, the topological properties of nodes have been characterized at a global level. While this approach has served to highlight important distinctions in processing, it is agnostic to the community organization of a network that may contain nodes with similar processing roles across distinct communities. The present observations serve to illustrate how nodes with similar roles can be present across distinct functional systems, and that these similarities predict functional responses across varying tasks.

The categorization of nodes based on the distinctions in their topological patterns relative to other nodes in the same functional system (community) revealed corresponding differences in connectional topology and task-evoked activity across individuals of different ages. Throughout the entire brain network, RSFC-defined non-connector nodes of each system exhibited greater task-evoked activity, relative to connector nodes. Importantly, this activation selectivity was most prominently observed in functional systems relevant to the processing demands of specific tasks that are mediated by those systems (i.e., the visual system in relation to the visual classification task, and the association systems in relation to the semantic task; Fig. 1*F*, Fig. 4). This specificity suggests that distinctions in connectional topology across nodes may be an important feature for establishing functional specialization, although we comment further on the direction of the influence below. Consistent with this general theme, recent work that has highlighted how structural connectivity can predict the activation of brain areas that are sensitive to processing specific stimulus categories (Saygin et al., 2016).

More broadly, the present work also aligns with emerging observations of other real-world networks, which have demonstrated how a node's topology is strongly linked with its functional specialization (Deng et al., 2012; Harriger et al., 2012; Turkina et al., 2016). An extension of this relationship predicts that disruptions in topological distinctions across nodes should result in a breakdown in their functional specialization. In our observations of brain networks, breakdowns in topological distinctions between brain areas that led to discrimination of connector and non-connector nodes were associated with corresponding reductions in the selective activation of non-connector nodes. We hypothesize that this reduction in selectivity underscores a reduction in functional specialization across different node types. This link between node topology and specialization exhibits interesting parallels in other network domains, such as networks of manufacturing (Turkina et al., 2016), thus bolstering support for the inferred relationship.

### Aging is accompanied by a breakdown in the differentiation of task signals over the brain network

The wide age range of our participant sample (20–89 years) allowed examination of differences in network connectional topology in relation to task-evoked brain activity. By using fMRI tasks that engaged distinct functional systems, we were able to highlight the specificity of the relationship between task activity and connectivity as a function of system type.

Presently, it is unclear whether the current observations are a consequence of the RSFC network constraining task activity, task activity sculpting the organization of RSFC networks, or a bidirectional relationship between the two. A number of studies have documented experience-dependent changes in specific RSFC patterns (Lewis et al., 2009; Albert et al., 2009; Harmelech et al., 2013). These observations, in combination with studies documenting age-related differences in RSFC have led to Hebbian-based hypotheses for the emergence of RSFC (Dosenbach et al., 2010; Wig et al., 2011). While we have largely framed our observations as a reflection of networks constraining task demands, it is equally possible that (age-dependent) differences in the specificity of areal recruitment may lead to differences in patterns of RSFC network patterns. One intriguing possibility is that a basic set of functional connections that are initially established from an individual's genetic and natal environment (Doria et al., 2010; Fransson et al., 2011) are recurrently sculpted into more (or less, as in senescence) specific topologies due to changes in neural activity associated with learning, environmental exposure, and basic neurophysiology (for review, see D'Esposito et al., 2003; Reuter-Lorenz and Park, 2014). In turn, the sculpted functional connections encourage functional responses that conform to these connections. Future studies using longitudinal data will enable us to explore how the brain's functional topology and activity influence one another over time.

While the direction of influence between topology and activity is currently unclear, the observed associations between age-related differences in RSFC topology and task activity indicate a coupling between a brain area's connectional properties and its function. By establishing a relationship between RSFC-defined topology and information processing of brain areas, the present results help to inform long-standing observations describing age-related reduction in the differentiation of task-evoked activation (Grady et al., 1994; Park et al., 2004). This dedifferentiation has been used to describe different age-related observations: (i) decreased specificity, where older adults exhibited less category specific activation in brain areas that were sensitive toward specific stimuli in younger adults (Park et al., 2004; Voss et al., 2008), (ii) and differential recruitment or decreased selectivity, where older adults activated additional brain areas compared to younger adults while performing the same tasks (Logan et al., 2002; Cabeza, 2002; Gutchess et al., 2005; Morcom et al., 2007). The present observations offer a possible account for the latter. Previous work has linked age-related differences in areal recruitment to a number of sources including behavioral differences (Logan et al., 2002; Hedden and Gabrieli, 2004; e.g., strategy) or a decreased ability of older adults to inhibit activations in brain areas that were irrelevant to the tasks (Logan et al., 2002; Lustig et al., 2003; Persson et al., 2007). Here, we suggest that one previously unrecognized source of the variance might be related to the topological differences observed in resting-state networks, which could impact the distinctions (differentiation) of task-evoked activity across brain areas. We propose that age-related differences in functional topology provide a unique and complementary network-based explanation for the widely observed effect of age-related dedifferentiation in functional activity.

Although it is beyond the scope of the present manuscript to investigate how other neurobiological variance may contribute to the observed effect, age-related changes in brain structure are a highly probable source (Reuter-Lorenz and Park, 2014). Reduced white matter integrity (Burzynska et al., 2010), white matter hyperintensities (Gunning-Dixon and Raz, 2000), and amyloid deposition (Dickson et al., 1992) have all been shown to both disrupt functional connections among highly connected areas (Buckner et al., 2009; Mormino et al., 2011) and impact brain activity (Nordahl et al., 2006; Sperling et al., 2009). These sources of degradation may encourage the reconfiguration of functional connections between previously less connected nodes of a brain network as observed here. It is currently unclear whether there is differential burden on connector versus non-connector nodes; the present results provide motivation for future inquiry regarding the age-related interaction between structural degradation and functional topology.

### Conclusion

The present results provide evidence that, within specific systems, RSFC-defined topological patterns of brain areas relate to selective (or differentiated) functional responses under varying stimulus- and task-processing demands. Age-related differences in brain network organization were used as a source of variation to further examine these relationships across individuals. The results revealed how distinctions in the topological patterns of brain networks may account for patterns of age-related reductions in the observed differentiation of brain activity. Although the present study focused on differences associated with healthy adult aging, the implication of the current findings extends to a more general understanding of the relationship between patterns of brain network topology and associated function.
